# Unethical psychiatrist’s communication toward patients serving a prison sentence – a case report

**DOI:** 10.1192/j.eurpsy.2023.1857

**Published:** 2023-07-19

**Authors:** L. Rossini Gajsak, L. Zibar

**Affiliations:** 1 Department of Biological Pscychiatry, Neuropsychiatric Hospital “Dr. Ivan Barbot”, Popovaca; 2 Department for Nephrology, Internal Clinic, University Hospital Merkur Zagreb, Department for Pathophysiology, Faculty of Medicine University Josip Juraj Strossmayer in Osijek; Croatian Medical Chamber, Head of Committee for Medical Ethics and Deontology, Zagreb, Croatia

## Abstract

**Introduction:**

An institutional ethical committee receives patients’ complaints regarding ethical side of physicians’ work, behavior and communication.

**Objectives:**

This case report presents an example of unethical communication by a psychiatrist toward patients serving a prison sentence, who sought a psychiatrist’s examination. The main objectives were to evaluate the physician’s insight into objections as well as adherence to ethical rules and regulations defined by ethical code, law and other related acts. Identity and other identification data have been modified in relation to the real case for confidentiality.

**Methods:**

The case of a written anonymous complaint to the institutional ethical committee and the psychiatrist’s statement on the patients’ accusations were taken as data source.

**Results:**

Prisoners seeking psychiatrist’s help complained about the behavior of their attendant prison psychiatrist and stated that he insulted and humiliated them, shouted at them, was telling them horrible things (like “you are going to dye slowly”), was talking about them behind their back, ignored their disturbances, listened to several patients at the same time and revealed their diagnoses in front of other patients. The physician denied all the accusations in his feedback report, but stated that there have been conflicts with these patients. He concluded that he wondered why he could not communicate with them more roughly, that there was too much work to do and a lack of time. Furthermore, he worked too much for a small fee and he did not respect their problems while they constantly asked for something, and thus that they made a burden to the health system. Finally, he would not have even respond to an anonymous report.

**Conclusions:**

In the presented case, there were many violations of ethical regulations and legal provisions. The psychiatrist made serious mistakes and misjudgments about numerous regulations of the Law on medical practice’s Article 21 regarding medical confidentiality (“Official Gazette” no. 117/08), Physician’s Oath Latest Version 2017, amended at the 68^th^ Assembly of the World Medical Association as well as the Croatian Code of Medical Ethics and Deontology („Official Gazette” no. 55/08, pages 1-7, Article 1, paragraphs 1,2,3,4,5,6, Article 2, paragraphs 1,2,14, Article 8, paragraphs 1,2, Article 9, paragraph 12, Article 10, paragraphs 1,2) regarding obligation to preserve the noble tradition of the medical profession by maintaining high standards of professional work and ethical behavior toward the patient, respecting the patient’s rights in physical and mental aspects, taking care of his personal dignity and securing a medical secret. Additional efforts must be made by various stakeholders in health care so that ethical postulates are more strongly embodied in everyday physician’s work, without arbitrary interpretations.

**Disclosure of Interest:**

None Declared

